# Problem Formulation Outweighs Loss-Function Choice in Deep-Learning Cervical Vertebral Maturation Staging: A Multi-Seed Study on an Imbalanced Public Benchmark

**DOI:** 10.3390/diagnostics16193183

**Published:** 2026-09-30

**Authors:** Nazlı Tokatlı

**Affiliations:** Department of Computer Engineering, Faculty of Engineering and Natural Sciences, Istanbul Health and Technology University, 34275 Istanbul, Turkey; nazli.tokatli@istun.edu.tr

**Keywords:** cervical vertebral maturation, deep learning, class imbalance, ordinal classification, orthodontics, cephalometry

## Abstract

(1) **Background**: Cervical vertebral maturation (CVM) staging on lateral cephalograms informs the timing of orthodontic treatment, and automated deep-learning approaches are widely studied. A recurring but under-examined obstacle is that public CVM datasets are severely imbalanced across the six stages. Using a public dataset and a rigorous eight-seed protocol, we quantify how strongly imbalance constrains performance, whether common imbalance-handling and ordinal-aware loss functions overcome it, and whether a clinically motivated three-group reformulation is more tractable than the native six-stage task. (2) **Methods**: Using the public Aariz dataset (1000 lateral cephalograms with expert CVM-stage labels; 700/150/150 train/validation/test, one radiograph per patient), we trained an ImageNet-pretrained ResNet-18 under four strategies: unweighted cross-entropy (baseline), class-weighted cross-entropy with balanced sampling, focal loss, and a rank-consistent ordinal (CORAL) head. Each configuration was trained with eight random seeds. Both tasks were evaluated over this multi-seed protocol, and sensitivity analyses examined the ordinal model’s sampling scheme and the focal-loss focusing parameter. Performance was assessed by accuracy, macro-F1, mean absolute error in stages (MAE), and quadratic weighted kappa (QWK), and compared across strategies with the Kruskal–Wallis test; secondary pairwise contrasts were Holm-corrected, and Wilson binomial confidence intervals (accuracy), seed-level confidence intervals (macro-F1), and analytically derived majority and frequency-weighted random classifiers were added as trivial reference baselines. Both the native six-stage task and a three-group scheme (pre-peak = CS1-3, peak = CS4, post-peak = CS5-6) were evaluated. (3) **Results**: On the six-stage task the extreme imbalance (CS1 *n* = 18 vs. CS5 *n* = 311 in training) produced poor minority-stage recognition (macro-F1 ≈ 0.22–0.25), with the rarest stages essentially unclassified even after imbalance handling. Reframing into three clinically meaningful groups roughly doubled macro-F1 (to ≈0.47–0.51) across all strategies. Critically, in the three-group setting no loss function significantly outperformed the others: across eight seeds the Kruskal–Wallis test was non-significant for every metric (macro-F1 *p* = 0.09, QWK *p* = 0.05, MAE *p* = 0.07, accuracy *p* = 0.41), and the unweighted baseline matched the best specialized loss on macro-F1 (0.507 ± 0.045 vs. ordinal 0.505 ± 0.044). In secondary pairwise comparisons the ordinal loss nominally exceeded the weighted and focal losses (uncorrected *p* = 0.04 and *p* = 0.01), but after Holm correction only the comparison with focal loss remained significant (adjusted *p* = 0.03), and no pairwise difference survived a conservative six-comparison Bonferroni bound; focal loss was the least stable. All strategies clearly exceeded trivial baselines on macro-F1 (majority classifier 0.259; frequency-weighted random ≈0.333), although the majority classifier’s accuracy (0.633) exceeded that of every trained model. (4) **Conclusions**: On this imbalanced public dataset, the dominant factor associated with CVM classification performance was the problem formulation rather than the loss function: a clinically grounded three-group scheme was far more tractable than six-stage classification, whereas specialized imbalance and ordinal losses did not reliably outperform a simple baseline. These findings, established under a fixed backbone, training budget, and a single public dataset, caution against assuming that loss-level fixes resolve CVM imbalance, and highlight data scale and problem formulation as the primary levers. Multi-dataset external validation is a necessary next step.

## 1. Introduction

Skeletal maturity assessment is central to orthodontic treatment planning, particularly for timing growth-modification interventions. The cervical vertebral maturation (CVM) method, which classifies maturation into six stages (CS1–CS6) from the morphology of the second to fourth cervical vertebrae on a lateral cephalogram, is widely used because it requires no additional radiographic exposure beyond the routine cephalogram [1,2]. However, manual CVM staging is time-consuming and subject to inter- and intra-observer variability: meta-analytic evidence places inter-observer agreement at a substantial but imperfect level (weighted kappa typically 0.6–0.8), with particular difficulty at intermediate stages [3,4,5]. This inherent label uncertainty both motivates automation and sets a realistic ceiling on achievable model performance.

Deep learning, and convolutional neural networks (CNNs) in particular, has been applied extensively to automated CVM staging, with reported six-stage accuracies varying widely across datasets and methods, from roughly 60–75% for image-only models to above 90% in some datasets [5,6,7,8,9,10,11,12,13,14,15]. A recurring and clinically important difficulty is class imbalance: the six CVM stages are rarely evenly represented in clinical samples, and minority stages are systematically harder to classify—a pervasive challenge across machine-learning applications [16,17]. Prior work has addressed this with strategies such as cervical-region cropping, custom architectures, and oversampling [7,8,12,18,19]. Nonetheless, comparisons of imbalance-handling strategies on a common, fully public benchmark remain limited, and the ordinal nature of CVM (the stages form an ordered sequence) is often not explicitly modelled.

This study addresses three questions on a public CVM-labelled dataset [20]: (i) how severely class imbalance constrains six-stage CVM classification; (ii) whether a clinically motivated three-group reformulation (pre-peak, peak, post-peak) improves tractability; and (iii) whether common imbalance-handling and ordinal-aware loss functions-class weighting, focal loss, and a rank-consistent ordinal formulation-outperform an unweighted baseline. To draw reliable conclusions given the modest dataset size, every configuration was evaluated over eight random seeds and compared with formal statistical tests, an unusually rigorous protocol for this task that guards against the single-run comparisons common in the CVM deep-learning literature. We deliberately hold the architecture and preprocessing fixed to isolate the effects of problem formulation and loss function.

## 2. Materials and Methods

### 2.1. Dataset

We used the publicly available Aariz dataset [20,21], released under a Creative Commons Attribution (CC-BY) license, which provides 1000 lateral cephalograms with expert CVM-stage annotations, split by the dataset authors into 700 training, 150 validation, and 150 test images. The 1000 cephalograms were collected from 1000 distinct patients aged 12 to 62 years [20]; because each patient contributes exactly one radiograph, patient-level independence between the training, validation, and test splits holds by construction. Per-image age and sex metadata are not distributed with the public dataset (see Limitations). Each image carries a single CVM stage label from CS1 to CS6. The dataset is markedly imbalanced: in the training split the stage counts were CS1 = 18, CS2 = 38, CS3 = 37, CS4 = 185, CS5 = 311, and CS6 = 111, a roughly seventeen-fold difference between the rarest and most frequent stage. The validation and test splits showed the same skew (e.g., only 5 CS1 images in each). No new patient data were collected; only the public dataset was analysed. We deliberately restricted the study to this single, well-characterized public benchmark and a single backbone: because our aim is to isolate the effect of problem formulation and loss function, holding the dataset and architecture fixed removes confounds that a multi-dataset or multi-backbone design would introduce. As the ground-truth CVM labels derive from expert visual staging, they carry the inherent observer uncertainty noted above (weighted kappa 0.6–0.8 in the literature [3,4]), which bounds the maximum achievable accuracy for any model trained on them.

For completeness and to be in line with data-transparency requirements, the provenance, expert validation, ethical status, and licensing of the dataset are detailed below.

**Source and access.** The Aariz dataset was published by Khalid et al. [20] (Scientific Data 2025;12:1336) and is deposited on Figshare (DOI: https://doi.org/10.6084/m9.figshare.27986417; accessed on 15 July 2026) [21]. It provides 1000 lateral cephalograms with CVM-stage annotations.

**Original data provenance.** The radiographs were curated from a larger pool of images acquired on seven different imaging devices, contributed by clinicians at Islamic International Dental College with the support of Riphah International University, Islamabad, Pakistan, as reported by the dataset authors.

**Expert validation.** CVM-stage labels were produced by expert orthodontists and reviewed by the dataset authors, with a full expert re-review of the dataset to correct annotation errors. Dataset-specific evidence underscores the intrinsic difficulty of CVM labelling: CVM-stage agreement between the two junior orthodontists was only 36.3%, after which the two senior orthodontists relabelled the dataset collaboratively, reaching 96.6% agreement (906 of 1000 images), with the remaining 94 disagreements adjudicated by expert orthodontists [20]. Because CVM staging is inherently subject to observer variability (weighted kappa 0.6–0.8 in the literature [3,4]), we treat these labels as the reference standard while acknowledging their intrinsic uncertainty.

**Ethics and anonymization.** The dataset was released as fully anonymized data, with all personally identifiable information removed in compliance with ethical guidelines and images used with patient consent as reported by the dataset creators. Secondary analysis of this de-identified public dataset did not require additional institutional review board approval.

**License compliance.** The dataset is publicly available under a Creative Commons Attribution (CC-BY) license. Our use complies with these terms; no images were redistributed, and the dataset is cited as required [20,21].

### 2.2. Classification Tasks

Two formulations were evaluated. The native six-stage task classifies each image as CS1–CS6. The three-group task collapses stages into pre-peak (CS1–CS3), peak (CS4), and post-peak (CS5–CS6), a grouping that reflects the principal clinical decision of whether a patient is before, at, or past the pubertal growth peak; merging of adjacent early stages has precedent in prior CVM deep-learning work [7]. Clinically, the Baccetti–Franchi–McNamara framework places the peak in mandibular growth in the interval around CS3–CS4 [1,2], so whether a patient is before, at, or past this interval is the primary determinant of the timing of growth-modification (e.g., functional-appliance) treatment; the three-group scheme maps directly onto this decision. Training-group counts were 93, 185, and 422 respectively—still imbalanced, but without the extremely small classes of the six-stage task.

### 2.3. Model and Training Strategies

All experiments used an ImageNet-pretrained ResNet-18 backbone [22], chosen for three reasons: residual CNNs of comparable depth have been repeatedly and successfully applied to CVM staging [14]; with only 700 training images, deeper backbones carry a higher risk of overfitting; and its computational efficiency made the full 72-run multi-seed protocol feasible; architecture optimization was not an aim of this study, and the backbone and all preprocessing were held fixed across strategies so that observed differences reflect only the loss function. Each cephalogram was loaded in grayscale, resized to 224 × 224 pixels, scaled to [0, 1], replicated across three channels to match the ImageNet-pretrained input [23], and normalized by subtracting 0.5 and dividing by 0.25. During training only, brightness augmentation [24] was applied with probability 0.5 by multiplying pixel intensities by a factor drawn uniformly from [0.8, 1.2] and re-clipping to [0, 1]; no augmentation was applied at test time. Models were trained for 30 epochs with the Adam optimizer (learning rate $1\times 10^{-4}$), batch size 16, on an Apple M2 Pro (Apple Inc., Cupertino, CA, USA) using the PyTorch [25] Metal Performance Shaders (MPS) backend.

Image quality control was inherited from the dataset curation process: the dataset authors selected the 1000 radiographs from a pool of approximately 3500 according to explicit inclusion and exclusion criteria under expert orthodontist review [20]. We applied no additional exclusions and used all 1000 images as distributed; beyond the resizing, intensity scaling, normalization, and brightness augmentation described above, no further preprocessing (cervical-region cropping, histogram equalization, or artifact removal) was performed, so that the reported results reflect the dataset in its publicly distributed form.

Four training strategies were compared, differing only in the loss function and sampling. (i) Baseline: standard unweighted cross-entropy with shuffled sampling. (ii) Weighted: class-weighted cross-entropy using square-root inverse-frequency weights, normalized to unit mean; for the three-group task these weights were approximately 1.38 (pre-peak), 0.98 (peak), and 0.65 (post-peak), paired with a weighted random sampler drawing minority examples more frequently. (iii) Focal loss [26] with focusing parameter γ = 2.0 and the same class weights and sampler. (iv) Ordinal: a rank-consistent (CORAL-style) head [27,28,29] that replaces the K-way softmax with K−1 binary threshold outputs (two for the three-group task, five for six stages), each predicting whether the true stage exceeds a given rank; the loss is the mean binary cross-entropy over these thresholds and the predicted class is the number of thresholds passed. The ordinal model used shuffled rather than weighted sampling, as weighted sampling was found to destabilize threshold learning in preliminary runs. Each of the four strategies was trained with eight random seeds (seeds 0–7), controlling PyTorch and NumPy random number generation, giving 32 training runs for the main three-group comparison (72 runs in total across all analyses, including the six-stage task and the sensitivity analyses of Section 3.3). The validation split was used to confirm convergence; no early stopping or per-strategy hyperparameter tuning was performed, and all reported metrics are computed on the held-out test split.

### 2.4. Evaluation

Performance was summarized by four metrics: overall accuracy; macro-averaged F1 (giving equal weight to each class, sensitive to minority-class failure); mean absolute error in stage units (MAE, penalizing predictions further from the true stage); and quadratic weighted kappa (QWK), a standard ordinal agreement measure that penalizes larger disagreements more heavily [30]. Macro-F1 was pre-specified as the primary metric, because it is the summary most sensitive to the minority-class failure that motivates this study. Macro-F1 was computed as the unweighted mean of per-class F1 scores; QWK used the standard quadratic weighting matrix with weights ${\left(i-j\right)}^{2}/{\left(K-1\right)}^{2}$. Results are reported as mean ± standard deviation over the eight seeds. Between-strategy differences were tested with the Kruskal–Wallis test across all four strategies for each metric (*n* = 8 per strategy), which was treated as the primary (omnibus) test; *p* < 0.05 was considered significant. Pairwise one-sided Mann–Whitney U tests on macro-F1 were restricted to three pre-specified secondary contrasts—the ordinal model versus each alternative—reflecting the directional hypothesis that the only rank-aware loss would improve macro-F1; the original submission stated this family incompletely, which is corrected here. These contrasts are reported with Holm–Bonferroni correction for the three-test family and, as a conservative bound, against a Bonferroni correction for all six possible pairs; given the non-significant omnibus tests, they are interpreted as secondary and exploratory.

Uncertainty was quantified in two complementary ways. Because every run is evaluated on the same fixed 150-image test split, seed-to-seed standard deviation reflects training stochasticity (weight initialization, mini-batch ordering, augmentation) rather than sampling variability of the test data; the sampling uncertainty of a single run’s accuracy was therefore quantified with Wilson 95% binomial confidence intervals at *n* = 150. In addition, 95% confidence intervals for mean macro-F1 across seeds were computed from the t distribution with 7 degrees of freedom (mean ± 2.365·SD/$\sqrt{8}$). A test-set bootstrap for macro-F1 would require stored per-image predictions, which were not retained (see Limitations). Two trivial reference classifiers were derived analytically from the test-split class frequencies (pre-peak/peak/post-peak = 15/40/95): a majority classifier that always predicts the most frequent group, and a frequency-weighted random classifier that predicts each group with probability equal to its empirical frequency.

Analyses used Python 3.14 and PyTorch 2.14.0, timm, scikit-learn-equivalent metric implementations verified against scipy, and scipy.stats for testing. Code is available from the corresponding author on reasonable request.

Two sensitivity analyses were performed on the three-group task. First, to address the fact that the ordinal (CORAL) model used shuffled rather than class-weighted sampling, the ordinal model was retrained with class-weighted sampling across all eight seeds and compared to the original. Second, because focal loss is sensitive to its focusing parameter, the focal-loss experiments were repeated at γ = 1, 2, and 3 (eight seeds each) and each was compared against the baseline. In addition, the six-stage task was run over the full eight-seed protocol (baseline and weighted strategies) rather than a single run, so that all reported results rest on multi-seed estimates.

## 3. Results

### 3.1. Six-Stage Task: Severe Imbalance Effects

**Figure 1:**
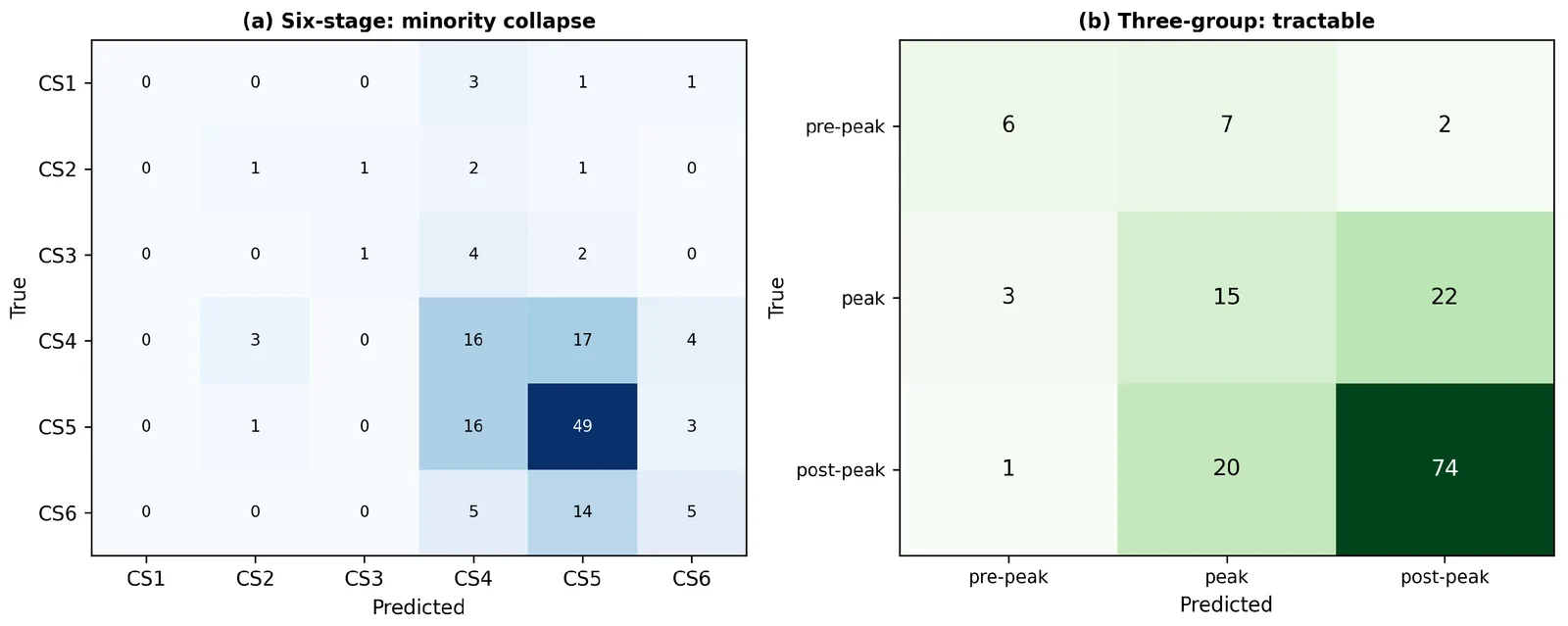
Confusion matrices contrasting (**a**) the six-stage task, where predictions collapse onto the majority stages CS4–CS5 and the rarest stages receive few or no correct predictions, and (**b**) the three-group task, where the confusion matrix is far better conditioned. Rows are true labels, columns predicted. Cell shading encodes the count printed in each cell (darker shades indicate larger counts) and carries no additional meaning.

We first evaluated the native six-stage task (CS1–CS6). The baseline unweighted model reached an overall accuracy of approximately 0.43 (0.427 ± 0.035 over eight seeds) but a macro-F1 of only about 0.22 (0.222 ± 0.025), exposing a large gap between overall accuracy and per-class performance that is characteristic of imbalanced classification. Inspection of the confusion matrix (Figure 1a) showed that predictions were concentrated on the two majority stages (CS4 and CS5): the rarest stages received few or no correct predictions, with CS1 and CS3 in particular frequently unclassified. Because overall accuracy is dominated by the majority stages, it substantially overstates clinical usefulness here; macro-F1, which weights each stage equally, is the more honest summary and reveals the failure.

Applying imbalance-handling (square-root inverse-frequency class weighting together with a matching weighted sampler) improved macro-F1 to approximately 0.25 and raised overall accuracy modestly (to about 0.44), and it partially revived some intermediate minority stages. However, it did not resolve the collapse of the extreme minority stages: with as few as 18 training and 5 test images for CS1, no loss-level adjustment could recover reliable performance, and per-stage F1 for CS1 and CS3 frequently remained at or near zero. Table 1 summarizes the six-stage results over eight seeds. These observations—a persistent floor on minority-stage recognition driven by data scarcity rather than by the training objective—directly motivated the clinically grounded three-group reformulation.

### 3.2. Three-Group Task: Strategy Comparison

**Figure 2:**
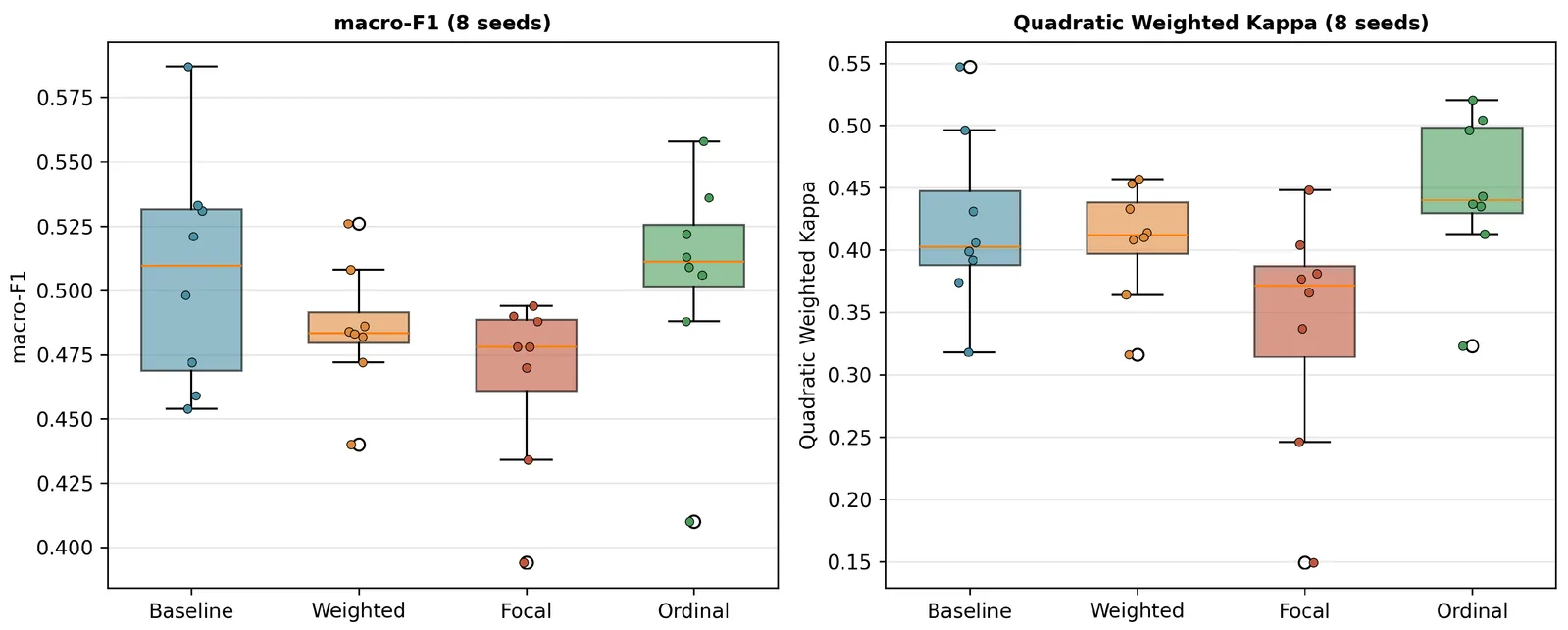
Distribution of macro-F1 and quadratic weighted kappa across eight seeds for the four training strategies in the three-group task. Boxes span the interquartile range; points are individual seeds. The strategies overlap substantially; focal loss shows the widest spread, including one low-QWK seed. Box colours serve only to distinguish the four strategies and encode no additional information.

Collapsing to three clinically meaningful groups substantially improved all metrics, roughly doubling macro-F1 relative to the six-stage task. Full three-group results over eight seeds are given in Table 2 and visualized in Figure 2. The central result is a negative one: no loss function significantly outperformed the others. The Kruskal–Wallis test was non-significant for every metric (macro-F1 H = 6.38, *p* = 0.09; QWK H = 7.74, *p* = 0.05; MAE H = 7.20, *p* = 0.07; accuracy H = 2.87, *p* = 0.41). Notably, the unweighted baseline achieved the highest mean macro-F1 (0.507 ± 0.045), statistically indistinguishable from the ordinal model (0.505 ± 0.044), while the ordinal model gave the best mean QWK (0.446 ± 0.063) and MAE (0.416 ± 0.046). In the secondary pairwise contrasts the ordinal loss nominally exceeded the weighted (uncorrected *p* = 0.04) and focal (uncorrected *p* = 0.01) losses, but not the baseline (*p* = 0.44); after Holm correction only the comparison with focal loss remained significant (adjusted *p* = 0.03; ordinal vs. weighted adjusted *p* = 0.08), and under a conservative Bonferroni bound for all six possible pairs no pairwise difference would remain significant (Section 2.4). Focal loss was the weakest and least stable strategy, with the widest seed-to-seed variance (QWK SD 0.096, including one seed at 0.15; Figure 2). In short, specialized imbalance and ordinal losses did not translate into a reliable advantage over a simple baseline on this task.

Two reference points contextualize these results. The majority classifier attains an accuracy of 0.633—higher than every trained model (0.591–0.617)—yet a macro-F1 of only 0.259; the frequency-weighted random classifier attains accuracy 0.482 and macro-F1 ≈ 0.333. All four trained strategies therefore clearly exceed the trivial baselines on macro-F1 (0.466–0.507) while offering no accuracy advantage over always predicting post-peak, which quantifies why accuracy is uninformative on this task and why macro-F1 is the primary metric. Consistent with this, Wilson 95% binomial confidence intervals for a single run’s accuracy at *n* = 150 are wide relative to the observed differences: baseline 0.608 [0.528–0.682]; weighted 0.617 [0.537–0.691]; focal 0.591 [0.511–0.666]; ordinal 0.598 [0.518–0.673]; majority 0.633 [0.553–0.706]. The maximum between-strategy accuracy difference (0.026) is far smaller than the interval half-width (≈0.077), so no accuracy difference among strategies is interpretable at this test-set size. Seed-level 95% confidence intervals for mean macro-F1 (t distribution, 8 seeds) were baseline 0.507 [0.469–0.545]; weighted 0.485 [0.464–0.506]; focal 0.466 [0.437–0.495]; and ordinal 0.505 [0.468–0.542]. These quantify uncertainty in the seed mean under training stochasticity and likewise overlap substantially across strategies.

**Figure 3:**
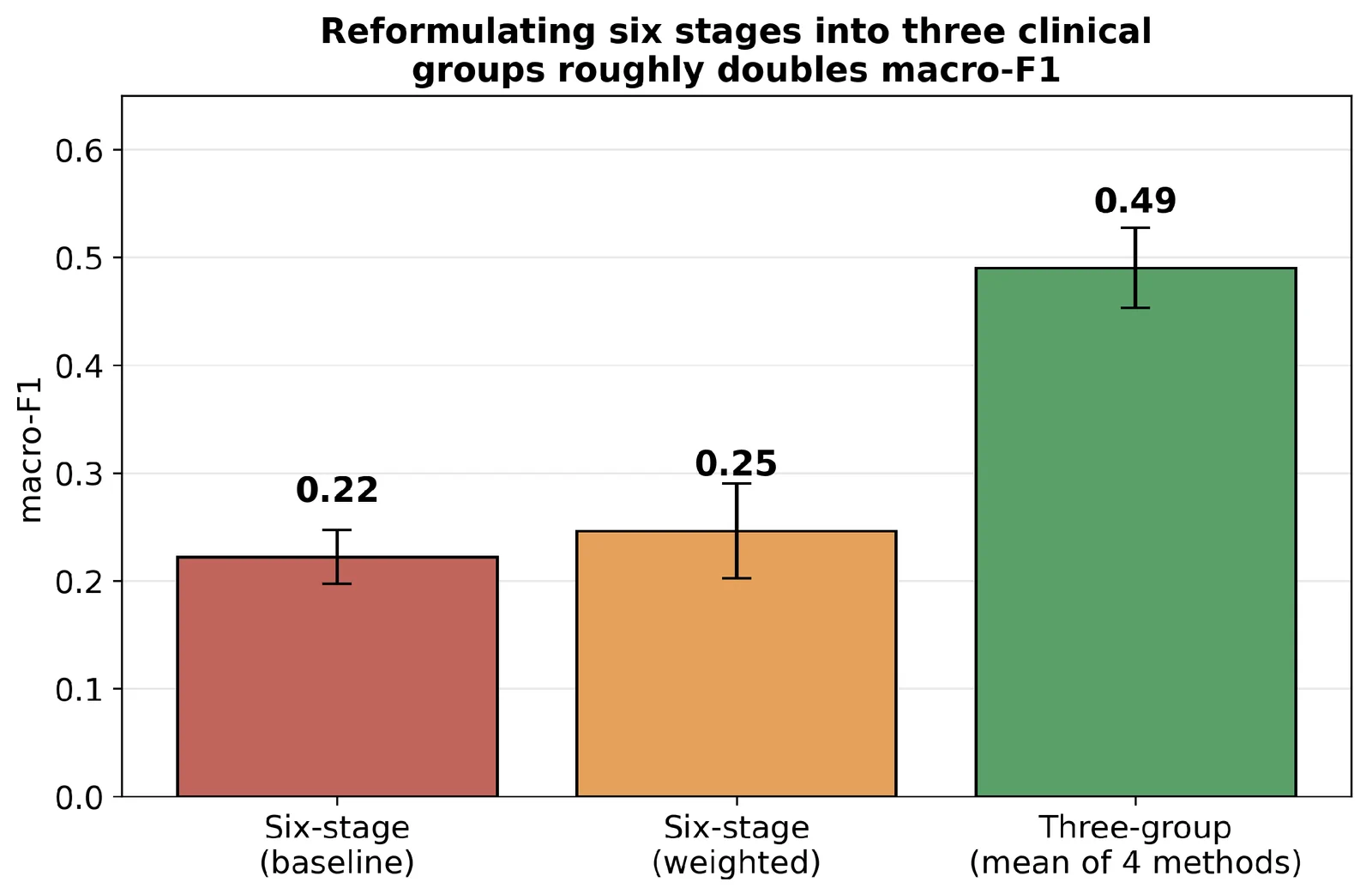
Macro-F1 for six-stage classification (baseline and weighted, eight seeds) versus the mean of four strategies in the three-group task (eight seeds; error bar = mean per-strategy SD). Reformulating the problem, rather than changing the loss, produces the largest gain. Bar colours serve only to distinguish the three bars and encode no additional information.

For reference, the best six-stage macro-F1 (≈0.25) was far below the three-group macro-F1 of all four strategies (0.47–0.51), quantifying the tractability gained by the clinical grouping (Figure 3). This formulation effect (≈0.25 to ≈0.50) dwarfs any difference between loss functions within the three-group task (all within ≈0.04 of one another).

### 3.3. Sensitivity Analyses

Two additional analyses tested whether the central negative result depended on specific design choices in the three-group comparison.

**Sampling scheme for the ordinal model.** In the main comparison, the ordinal (CORAL) model used shuffled rather than class-weighted sampling, because in preliminary runs weighted sampling destabilized threshold learning; this meant the ordinal strategy differed from the others in both loss and sampling. To test whether this confound affected the result, we retrained the ordinal model with class-weighted sampling across all eight seeds and compared it to the shuffled-sampling version. The two configurations did not differ significantly on macro-F1 (weighted 0.521 ± 0.033 vs. shuffled 0.505 ± 0.044; Mann–Whitney *p* = 0.44), MAE (*p* = 0.06), or QWK (*p* = 0.07); a small difference in accuracy reached nominal significance (0.647 ± 0.028 vs. 0.598 ± 0.047; *p* = 0.02). Thus the sampling choice did not materially change the ordinal model’s standing on the primary metric, and the conclusion that no loss function dominates is not an artifact of the sampling difference.

**Focal-loss focusing parameter.** Because focal loss is known to be sensitive to its focusing parameter γ, we repeated the focal-loss experiments at γ = 1, 2, and 3 (eight seeds each) and compared each against the unweighted baseline. At no value of γ did focal loss significantly exceed the baseline on macro-F1 (γ = 1: 0.472 ± 0.041, *p* = 0.93; γ = 2: 0.466 ± 0.035, *p* = 0.95; γ = 3: 0.479 ± 0.044, *p* = 0.92; one-sided Mann–Whitney), and in every case the point estimate was at or below the baseline (0.507 ± 0.045). The finding that focal loss provides no advantage in this setting is therefore robust to the choice of γ.

Together, these analyses indicate that the primary conclusion—that problem formulation, rather than the loss function, is the dominant factor associated with performance—is stable with respect to the ordinal model’s sampling scheme and the focal-loss focusing parameter.

## 4. Discussion

**Principal findings.** This study yields three findings. First, six-stage CVM classification on a severely imbalanced public dataset is unreliable for minority stages, which remain essentially unclassified despite imbalance handling. Second, a clinically grounded three-group scheme (pre-, at-, and post-peak) is markedly more tractable, roughly doubling macro-F1. Third, and central to this work, once the three-group formulation is adopted the choice of loss function does not matter in a statistically detectable way: across eight seeds no specialized loss significantly outperformed an unweighted baseline (Kruskal–Wallis *p* ≥ 0.05 for all metrics), and the baseline matched the best method on macro-F1. After multiplicity correction, the ordinal loss retained only a single nominal pairwise edge (over focal loss), and none under a conservative six-comparison bound. The dominant lever was thus the problem formulation, not the training objective. We emphasize that this is a controlled comparison by design: fixing the dataset, backbone, and training budget isolates the loss function as the only varying factor within the three-group comparison (with the ordinal model’s sampling scheme additionally addressed by the sensitivity analysis of Section 3.3), so the null result is a clean statement about loss functions specifically.

**Interpreting the formulation effect.** The six-to-three regrouping is a compound intervention: it simultaneously reduces the number of classes, merges precisely those adjacent stages that are hardest to distinguish, and lowers—but does not remove—the imbalance ratio (from roughly 17:1 across six stages to approximately 4.5:1 across three groups). These components cannot be disentangled in the present design, and we therefore do not attribute the formulation effect to imbalance reduction alone; a controlled experiment varying the imbalance ratio at a fixed number of classes (e.g., by majority-class subsampling) would be required for that attribution and is identified as future work. Accordingly, we describe problem formulation—the joint intervention—as the dominant factor associated with performance in this dataset and setting; the revised title reflects this calibration. Relatedly, the majority classifier’s higher accuracy alongside its far lower macro-F1 (Section 3.2) illustrates that under this class distribution accuracy rewards degenerate constant prediction, and only class-balanced metrics register genuine discriminative ability: the trained models’ value lies in recovering the pre-peak and peak groups, which the majority classifier ignores entirely.

**Relation to prior work.** Reported six-stage CVM accuracies vary widely, from roughly 60-75% for image-only models to over 90% in some datasets, differences driven largely by dataset composition, sample size, and preprocessing [5,6,7,8,10,14,15]. Our six-stage figures sit at the lower end, reflecting the specific imbalance of this public dataset and our deliberate use of a single untuned backbone rather than a bespoke architecture or cervical-region cropping. ResNet-family backbones remain a common and effective choice in maxillofacial deep-learning applications more broadly [31]. Prior work has consistently identified imbalance as a central obstacle, addressing it with oversampling or region isolation [7,12,19], and adjacent-stage merging has precedent [7]. Our contribution is to show, with a multi-seed statistical protocol, that on this dataset the commonly recommended loss-level remedies do not yield a statistically reliable improvement over a plain baseline—a caution directly relevant to the many single-run comparisons in the CVM literature, where apparent method rankings may reflect seed variance rather than genuine differences. Indeed, our own preliminary three-seed evaluation had suggested an ordinal advantage in macro-F1 that disappeared under eight-seed evaluation, illustrating the point. The dataset itself functions as a shared community benchmark: it was introduced through the CEPHA29 challenge at IEEE ISBI 2023, in which 29 international teams participated, and its publication has already accrued multiple citations [20].

The robustness of our central result is reinforced by the sensitivity analyses. The negative finding does not depend on the ordinal model’s sampling scheme, nor on the focal-loss focusing parameter, and it holds for the six-stage task under the full eight-seed protocol as well. This consistency across design choices strengthens the interpretation that performance is associated primarily with problem formulation rather than the training objective or its incidental configuration.

**Label noise as a performance ceiling (hypothesis).** A plausible—though in this dataset not directly verified—contributor to the modest absolute performance and to the indistinguishability of loss functions is the inherent uncertainty of CVM ground truth. Reported inter-observer agreement for manual CVM staging is substantial but bounded (weighted kappa 0.6–0.8), and is poorest precisely at the intermediate stages that our three-group scheme merges [3,4,5]. Dataset-specific evidence points the same way: junior-orthodontist CVM agreement in this dataset was only 36.3%, with final labels produced by collaborative senior relabelling (96.6% agreement) and expert adjudication of 94 residual disagreements [20]. When labels themselves disagree at such rates, differences between loss functions of a few macro-F1 points may fall within the noise floor set by the annotations, and no training objective can be expected to exceed the reliability of its supervision. We present this ceiling argument explicitly as a hypothesis rather than an established mechanism, since inter-observer variability was not independently quantified in this study. This reframes our null result: rather than a failure to find the right loss, it suggests the achievable headroom on this task is constrained more by label quality and data scale than by the choice of training objective.

**Clinical interpretation.** The three-group formulation aligns with the principal clinical question in growth-modification timing: whether a patient is before, at, or past the pubertal peak. In our experiments, three-group accuracy of roughly 0.60–0.62 and QWK around 0.43–0.45 indicate moderate agreement, well below the substantial inter-observer agreement reported for human experts (weighted kappa 0.6–0.8) [4,5,7]. The dominant residual confusion is between the peak and post-peak groups (Figure 1b); an erroneous post-peak prediction for a patient who is in fact at peak would risk missing the optimal window for functional-appliance treatment, the costliest clinical error in this setting. At this performance level the system is therefore suitable at most as a screening or decision-support prior whose outputs are verified by a clinician, and not for autonomous staging, pending further validation.

**Limitations.** Several limitations qualify these findings. The study uses a single public dataset and a single backbone. This was a deliberate design choice to isolate the loss-function effect, but it necessarily limits external generalizability: the specific magnitudes reported here, and the loss-function null result, require confirmation on independent datasets and populations before being treated as general. No conclusion of this study should be read as established beyond the evaluated dataset, backbone, and training regime. While eight seeds provide reasonable stability, the non-significant differences between losses should not be over-interpreted as proven equivalence, since modest true effects could go undetected at this sample size (a Type II risk we state explicitly rather than claim equivalence). Because all seeds share one fixed test split, seed-to-seed variability quantifies training stochasticity (initialization, sampling order, augmentation) rather than sensitivity to data sampling, and the single-run binomial uncertainty of accuracy at *n* = 150 (≈±0.077) exceeds all observed between-strategy accuracy differences; a test-set bootstrap for macro-F1 was not performed because per-image predictions were not retained, and seed-level t-based intervals are reported instead. The six-to-three regrouping is a compound intervention whose components (class count, adjacent-stage merging, imbalance ratio) are confounded in this design, and residual imbalance of approximately 4.5:1 persists in the three-group task. The ordinal model’s sampling scheme differed from that of the weighted and focal strategies; although the sensitivity analysis of Section 3.3 indicates this did not drive the results, it remains a design asymmetry. Per-image age and sex metadata are not distributed with the public dataset, so demographic representativeness could not be assessed beyond the documented 12–62-year age range [20]. The dataset’s severe imbalance limits minority-stage evaluation (as few as five test images per rare stage), and stage-specific performance estimates for CS1–CS3 should be interpreted with corresponding caution. We did not perform cervical-region cropping—which prior work suggests can help—because the dataset’s landmark set does not include cervical vertebral points, so region isolation would require an additional detection step. Ground-truth CVM staging is itself subject to observer variability, which we did not quantify. The absence of per-strategy hyperparameter tuning is a further scope condition: the null result is established for a fixed, shared training regime, and tuned learning rates or longer schedules could in principle alter the ranking, although the γ sensitivity analysis (Section 3.3) indicates robustness for the focal loss specifically. Most importantly, no external or cross-dataset validation was performed; whether the formulation effect and the loss-function null result generalize to other datasets and populations remains to be established.

**Future work.** The clearest priority is external, cross-dataset validation with an independently sourced CVM dataset, which would test whether both the formulation effect and the loss-function null result hold across populations and imaging devices. A second priority is a controlled decomposition of the formulation effect: varying the imbalance ratio at a fixed number of classes (e.g., by majority-class subsampling) to separate the contributions of class count, adjacent-stage merging, and imbalance. Complementary directions include cervical-region cropping with a dedicated detection step, evaluation of additional backbones and larger models, and quantifying inter-observer variability as a human reference.

## 5. Conclusions

On a severely imbalanced public cephalometric dataset, the dominant factor associated with automated CVM classification performance was the problem formulation rather than the loss function. Six-stage classification was unreliable for minority stages, whereas a clinically motivated three-group formulation (pre-peak, peak, post-peak) roughly doubled macro-F1. Under a rigorous eight-seed protocol with multiplicity-corrected statistics, specialized imbalance-handling and ordinal losses did not reliably outperform a simple baseline, and all strategies exceeded trivial reference classifiers only on class-balanced metrics, not on raw accuracy. These conclusions are established for a single public dataset, a single backbone, and a fixed training regime; these results caution against assuming that loss-level adjustments resolve CVM class imbalance and identify problem formulation, data scale, and external validation as the primary levers for progress.

## Figures and Tables

**Table 1:** Six-stage CVM classification on the Aariz test split (eight seeds, mean ± SD). Even with imbalance handling, macro-F1 remained low and the rarest stages (CS1–CS3) were largely unclassified, reflecting extreme class scarcity. Differences between the two strategies were not statistically significant on any metric (Mann–Whitney, all *p* > 0.26).

Strategy (6-Stage)	Accuracy	Macro-F1	MAE	QWK
Baseline (CE)	0.427 ± 0.035	0.222 ± 0.025	0.775 ± 0.040	0.254 ± 0.052
Weighted CE + sampler	0.443 ± 0.024	0.246 ± 0.044	0.759 ± 0.044	0.298 ± 0.083

**Table 2:** Three-group CVM classification (pre-peak/peak/post-peak) on the Aariz test split. Mean ± SD over eight random seeds. Higher is better for accuracy, macro-F1 and QWK; lower is better for MAE. Kruskal–Wallis tests found no significant between-strategy difference for any metric (all *p* ≥ 0.05). The two reference rows are trivial classifiers derived analytically from the test-split class frequencies (15/40/95); their QWK is 0 by construction (constant prediction) and 0 in expectation under independence, respectively.

Strategy	Accuracy	Macro-F1	MAE	QWK
Baseline (CE)	0.608 ± 0.040	0.507 ± 0.045	0.436 ± 0.045	0.420 ± 0.072
Weighted CE	0.617 ± 0.028	0.485 ± 0.025	0.426 ± 0.034	0.407 ± 0.047
Focal loss	0.591 ± 0.024	0.466 ± 0.035	0.463 ± 0.032	0.339 ± 0.096
Ordinal (CORAL)	0.598 ± 0.047	0.505 ± 0.044	0.416 ± 0.046	0.446 ± 0.063
Majority classifier (reference)	0.633	0.259	0.467	0.000
Freq.-weighted random (reference)	0.482	≈0.333	0.644	0.000

## Data Availability

The Aariz dataset [20] is publicly available under a Creative Commons Attribution (CC-BY) license via the Figshare repository (DOI: https://doi.org/10.6084/m9.figshare.27986417.v1) [21]. No new data were generated in this study. Analysis code is available from the corresponding author on reasonable request.
